# Inter-Rater Reliability of Scoring Systems for Abomasal Lesions in Quebec Veal Calves

**DOI:** 10.3390/ani13101664

**Published:** 2023-05-17

**Authors:** Laura Van Driessche, Gilles Fecteau, Julie Arsenault, Léa Miana, Younes Chorfi, Marianne Villettaz-Robichaud, Pierre Hélie, Sébastien Buczinski

**Affiliations:** 1 Department of Clinical Science, Faculty of Veterinary Medicine, Université de Montréal, St-Hyacinthe, QC J2S 2M2, Canada; 2 Department of Veterinary Biomedicine, Faculty of Veterinary Medicine, Université de Montréal, St-Hyacinthe, QC J2S 2M2, Canada; 3 École Nationale Vétérinaire de Toulouse (ENVT), 31076 Toulouse, France; 4 Department of Pathology and Microbiology, Faculty of Veterinary Medicine, Université de Montréal, St-Hyacinthe, QC J2S 2M2, Canada

**Keywords:** abomasal lesions, veal calves, scoring system, reliability

## Abstract

**Simple Summary:**

Abomasal lesions are considered to be an important health issue in cattle, especially in milk-fed (or white) veal calves. Using a reliable scoring system to describe abomasal lesions can help in determining the possible risk factors in order to prevent this problem. The aim of this study was to determine the inter-rater reliability of scoring systems used for abomasal lesions. Additionally, macroscopic lesions were compared with histological lesions.

**Abstract:**

The objective of this study was to determine the inter-rater reliability of current scoring systems used to detect abomasal lesions in veal calves. In addition, macroscopic lesions were compared with corresponding histological lesions. For this, 76 abomasa were retrieved from veal calves in a slaughterhouse in Quebec and scored by four independent raters using current scoring systems. The localisations of the lesions were separated into pyloric, fundic, or torus pyloricus areas. Lesions were classified into three different types, i.e., erosions, ulcers, and scars. To estimate the inter-rater reliability, the coefficient type 1 of Gwet’s agreement and Fleiss *κ* were used for the presence or absence of a lesion, and the intra-class correlation coefficient was used for the number of lesions. All veal calves had at least one abomasal lesion detected. Most lesions were erosions, and most of them were located in the pyloric area. Overall, a poor to very good inter-rater agreement was seen for the pyloric area and the torus pyloricus regarding the presence or absence of a lesion (Fleiss *κ*: 0.00–0.34; Gwet’s AC1: 0.12–0.83), although a higher agreement was observed when combining all lesions in the pyloric area (Fleiss *κ*: 0.09–0.12; Gwet’s AC1: 0.43–0.93). For the fundic area, a poor to very good agreement was also observed (Fleiss *κ*: 0.17–0.70; Gwet’s AC1: 0.90–0.97). Regarding the inter-rater agreement for the number of lesions, a poor to moderate agreement was found (ICC: 0.11–0.73). When using the scoring system developed in the European Welfare Quality Protocol, a poor single random rater agreement (ICC: 0.42; 95% CI: 0.31–0.56) but acceptable average random rater agreement (ICC: 0.75; 95% CI: 0.64–0.83) was determined. Microscopic scar lesions were often mistaken as ulcers macroscopically. These results show that the scoring of abomasal lesions is challenging and highlight the need for a reliable scoring system. A fast, simple, and reliable scoring system would allow for large scale studies which investigate possible risk factors and hopefully help to prevent these lesions, which can compromise veal calves’ health and welfare.

## 1. Introduction

Abomasal lesions are a well-known problem in the cattle industry. In milk-fed veal calves, a high prevalence of abomasal lesions from 73% to even 100% was reported in Europe [1,2]. Considering the economic impact associated with these lesions [3], a proper understanding of this issue is crucial. Multiple risk factors are associated with abomasal lesions in calves [4], which makes prevention challenging. Also, a definitive ante mortem diagnosis remains difficult. In severe cases, when lesions perforate the abomasum wall, a localised or generalised peritonitis can occur, leading to high mortality rates [3,5,6]. However, until a lesion perforates the abomasal wall, clinical signs are not specific [7,8], and a conclusive diagnosis is only possible post-mortem [9,10,11].

Different scoring systems are used at the slaughterhouse to describe abomasal lesions. Most commonly, three types of lesions are described: erosions, ulcers, and scars [4,12]. Erosions are characterized by a superficial damage of the mucosa, leaving the lamina muscularis mucosae intact [9,12,13]. When the latter is perforated and the submucosa is affected, an ulcer is formed [9,12,13]. Scars are considered to be either healed or chronic ulcers, making a star-shaped image caused by the fibrous contractions of the mucosa [8,12] or with the presence of more consistent granular tissue. This is in contrast with erosions, where no scars are formed after healing since the regeneration is epithelial [4].

A second method of scoring is to obtain an overall number (ranging from 0 to 24) based on a weighted sum of the number (ranging from 0 in the absence of a detected lesion to 4 if 4 or more lesions are observed) of small (<0.5 cm^2^, score 1), medium (0.5–1.0 cm^2^, score 2) or large (>1.0 cm^2^, score 3) lesions [2,14].

Although these scoring systems are widely used, their inter-rater reliability, which reflects the variation between two or more raters who measure the same subjects, has never been reported. This information is critical to selecting the best-performing scoring system of abomasal lesions for further risk factor investigation or surveillance of the condition. Therefore, the objectives of this study were (1) to determine the inter-rater reliability of the current scoring systems used to detect abomasal lesions in calves, and (2) to compare the macroscopical typing of lesions with histological examination.

## 2. Material and Methods

### 2.1. Sample Size Calculation and Rater Selection

This research was conducted according to the guidelines for reporting reliability and agreement study [15]. No data were available in the literature regarding the inter-rater agreement and reliability of abomasal scoring systems in veal calves. The sample size was determined based on an expected lesion prevalence of 70% and the ability to detect true kappa reliability coefficients greater than 0.4, 0.5, and 0.6. Different scenarios were developed, taking into account the presence versus absence of a specific lesion (binary classification) using a freely available software (package irr [16], argument N.cohen.kappa; R [17]). A sample size of 70 abomasa was determined as adequate for all scenarios, with a maximal Type I error (α) set at 5% and Type-II error (β) set at 20%. These scenarios are robust regarding lesion prevalence, as this sample size was also suitable for a prevalence of lesions varying from 10% to 90%.

To evaluate the inter-operator reliability characteristics of the scoring systems, a minimum of 3 different operators were considered necessary. Therefore, 4 different raters with various levels of experience were enrolled in the study. Two raters were unexperienced veterinary students based in a veterinary faculty located either in St-Hyacinthe, Canada, or in Toulouse, France. The two other raters were senior and experienced; one was a DVM, PhD in veterinary science and one was a DVM, MSc, DACVIM, and professor in veterinary medicine. A brief 15 min explanation was given to each rater individually by an experienced person, clarifying different types of lesions and their localisations of the abomasa. This minimal training period was considered as a basic training session that could easily be applied for future use by slaughterhouse workers.

### 2.2. Data Collection

The abomasa were collected from 27 June to 11 July 2022 in a large veal slaughterhouse in Saint-Germain de Grantham, central Quebec, Canada, with an average slaughter capacity of 1200 white veal calves per week. For 7 days, between 10 to 15 abomasa per day were retrieved from white veal calves raised for meat production. During the slaughtering process, the abomasum was separated from the intestinal tract by experienced technicians working in the packing plant as a standard operation process. Immediately after separation, the organs were rinsed with tap water, collected in a box, and transported for a maximum of 40 min at room temperature to the Faculty of Veterinary Medicine in St-Hyacinthe (Quebec, QC, Canada). Upon arrival at the necropsy laboratory, the abomasa were opened along the greater curvature from the omasoabomasal orifice to the duodenum and rinsed a second time with tap water before examination. Each abomasum was numerically identified and photographically documented. Since the torus pyloricus is considered to be a predilection site for lesions, only abomasa that were complete (i.e., presence of the torus pyloricus) were kept for the study.

### 2.3. Macroscopical Examination of the Lesions

**Figure 1:**
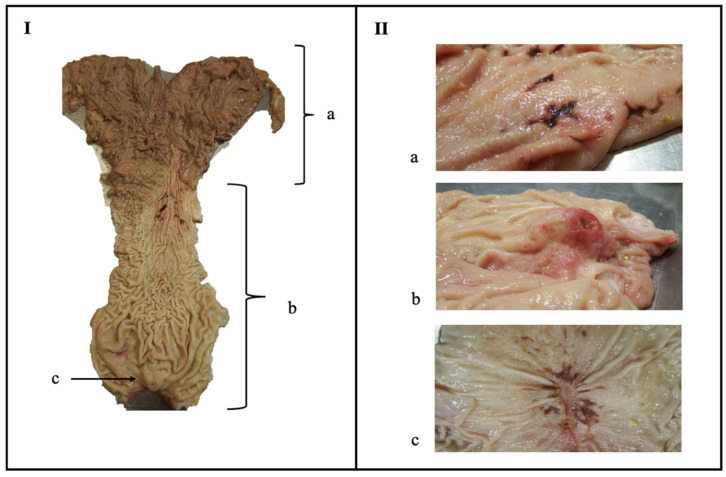
(**I**): Abomasum of a veal calf, separated as the fundus or fundic area (a), the pyloric area (b), and the torus pyloricus (c). (**II**): Types of lesions, classified as an erosion (a), ulcer (b), or scar (c).

Anatomical localisation and classification of the lesions were performed as shown in Figure 1. Briefly, erosions were characterized by superficial damage of the internal abomasal mucosa [9,12,13], whilst an ulcer showed more of a crater image and thus a deeper lesion since the submucosa was also affected [9,12,13]. Scars were considered chronic or healed ulcers, making a star-shaped image caused by the fibrous contraction [8,12] or with the presence of more consistent granular tissue. The localisation, type, and size of each lesion were noted. The size was measured in millimeters as length and width for square shapes, and as diameter for round shapes.

### 2.4. Histology

Tissue samples (1 × 1 cm) harboring a single macroscopical lesion were collected from multiple abomasa, distributed over the various types of lesions (60 samples in total). The samples were fixed by immersion in 10% buffered formalin for a period from 3 days to 2 weeks, embedded in paraffin blocks, and, afterwards, cut into 5 µm thick slices. All tissue sections were stained separately using hematoxylin-eosin and classified as erosion, ulcer, or scar by a board-certified anatomic pathologist (P. Hélie) who was blinded to the scoring system. Ulcers were differentiated from erosions based on the perforation of the lamina muscularis mucosae [9,12,13] and a scar was distinguished by the presence of variably mature granulation tissue [8,12].

### 2.5. Statistical Analysis

All data from the scoring systems were stored in an Excel file (Excel 2016, Microsoft Corp., Redmond, WA, USA). Analyses were performed using the open-source R software v 4.3.3 (https://www.r-project.org/ (accessed on 8 September 2022)). The detailed coding script can be found in the Supplementary Data. Scores were calculated per rater and per abomasum in accordance with the Welfare Quality Consortium protocol for veal calves, 2009 [2]. Only lesions in the pyloric area and torus pyloricus were taken into account. The surface of each lesion was estimated based on the size measurements of the rater, and was classified as follows: small (<0.5 cm^2^), medium (0.5–1.0 cm^2^), and large (>1 cm^2^). The number of lesions was set to 0 when no lesion was present and was truncated to 4 when 4 or more lesions were present. An overall score (between 0 and 24) was then calculated according to the following formula:

(1) $$\left(number\;of\;small\;lesions\times 1\right)+\left(number\;of\;medium\;lesions\times 2\right)+\left(number\;of\;large\;lesions\times 3\right)$$

To determine the overall inter-rater agreement for multiple raters, both Fleiss *κ* and Gwet’s agreement coefficient type 1 (AC1) were used for the presence or absence of a lesion in a particular region of the abomasum. Fleiss *κ* shows the average pairwise agreement between raters, averaged over all rater’s pairs and specimens, whilst Gwet’s AC1 demonstrates the chance-corrected agreement coefficient [18,19]. The latter is more stable than the Fleiss *κ* and is recommended when the prevalence of the studied outcome diverges from 0.5 [20]. The following guidelines were used for interpretation as previously reported [21]: poor agreement for values below <0.20; slight agreement for values between 0.21 and 0.40; moderate agreement for values between 0.41 and 0.60; good agreement for values between 0.61 and 0.80; and very good agreement for values between 0.81 and 1.00.

To verify if one particular rater substantially influences the outcome, the raw percentage of agreement (Pa), Cohen’s *κ*, and Gwet’s AC1 were used to compare pairs of raters. The Pa is defined as the number of abomasa for which 2 raters agreed divided by the total number of abomasa scored. According to the guidelines, a minimum of 0.75 is necessary to be considered acceptable [22,23]. Agreement beyond chance was obtained using Cohen’s *κ* between the pairs of raters [24]. The following guideline was used for interpreting Cohen’s *κ* [21]: poor agreement for values below <0.20; slight agreement for values between 0.21 and 0.40; moderate agreement for values between 0.41 and 0.60; good agreement for values between 0.61 and 0.80; and very good agreement for values between 0.81 and 1.00.

The intra-class correlation coefficient (ICC) was used to calculate the inter-rater agreement for two quantitative variables [19,25]: the number of the same kind of lesions in the same region and the score system developed by Brscic et al. [2]. The ICC reflects both the degree of correlation and the agreement between measurements, and was interpreted as follows [26]: ICC ≤ 0.5 = poor indicator of reliability; 0.5 < ICC ≤ 0.75 = moderate reliability; 0.75 < ICC ≤ 0.9 = good reliability; and >0.9 = excellent reliability. A ‘two-way random effect’ model was used [26] for the analyses. For the type of lesion, the type ‘single rater/measurement’ and definition ‘absolute agreement’ were chosen [27], also known as ICC (2,1) [25]. The type ‘average rater/measurement’ or ICC (2,k) [25] was used for the scoring system described by Brscic et al. [2].

## 3. Results

### 3.1. Prevalence of Lesions

**Figure 2:**
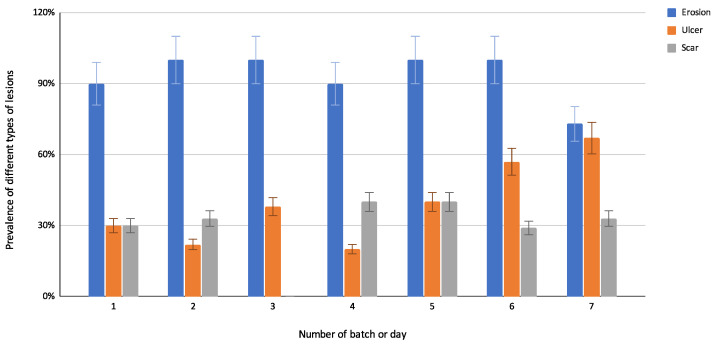
Prevalence (%) of different types of macroscopical lesions (erosion, ulcer, and scar) in 76 abomasa of veal calves in Quebec, separated per day or batch (from 1 to 7).

In total, 81 abomasa were retrieved from the slaughterhouse. Since only complete abomasa with the presence of a torus pyloricus were considered in the study, 76 abomasa (94%) were further investigated. The prevalence, median, and range of the number of macroscopic lesions per location and type based on the classification of one experienced rater can be found in Table 1. The most prevalent lesion type was erosion, and the most prevalent location of the lesions was the pyloric area. All abomasa showed at least one lesion, leading to a prevalence of 100% of lesions in abomasa retrieved from veal calves from a slaughterhouse in Quebec. The distribution of different types of lesions that were present in the abomasa per day can be found in Figure 2. Also, the most prevalent types of lesions every day were erosions, followed by ulcers and scars. A difference in the prevalence of lesions from the abomasa can be noticed depending on the day and, thus, the batch of animals that was present in the slaughterhouse.

### 3.2. Interrater Reliability

The Fleiss *κ*, Gwet’s AC1, and ICC for the four different raters assessing the abomasal lesions of 76 abomasa can be found in Table 2. Unsurprisingly, with the high prevalence settings, Gwet’s AC1 was higher than Fleiss *κ*. Overall, a poor to very good agreement was seen for the pyloric area and torus pyloricus, although a higher agreement was observed when combining lesions in the pyloric area. For the fundic area, a poor to very good agreement was also obtained regarding the presence or absence of a lesion (Fleiss *κ* and Gwet’s AC1), and a poor to moderate agreement was obtained regarding the number of lesions (ICC). Regarding the scoring system used by Brscic et al. [2], a single random rater agreement or ICC (2,1) of 0.42 (95% CI: 0.31–0.56) was obtained. For the average random rater agreement or ICC (2,k), a value of 0.75 (95% CI: 0.64–0.83) was determined. The median score (interquartile range), calculated from the data of the experienced observer, was 4 (4–8).

The raw percentage of agreement (Pa), and Cohen’s *κ* and Gwet’s agreement coefficient type 1 (AC1) for each pair of raters can be found in Table 3. Overall, the highest average Pa and AC1 could be found between rater 2 and 4, who are senior researchers. Besides the agreement between raters 1 and 3 and raters 1 and 4, the Pa was considered to be acceptable. Concerning the Cohen’s *κ*, poor to slight agreement was seen for the average between pairs, and a moderate to good agreement was seen for Gwet’s AC1.

### 3.3. Comparison with Histological Examination

**Figure 3:**
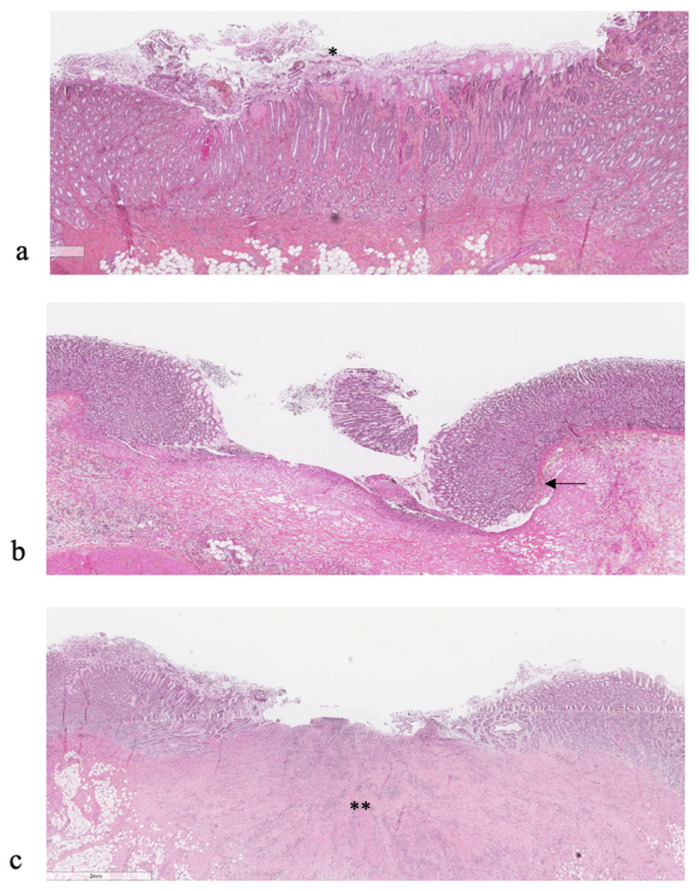
Histological presentation of (**a**) an erosion, (**b**) ulcer, and (**c**) scar in abomasa of veal calves. Only the mucosa (*) is damaged with erosions, in comparison with ulcers and scars where the lamina muscularis mucosae is penetrated (arrow). Additionally, in scars, an excessive amount of granular tissue (**) is found.

In total, 60 tissue samples with one macroscopic lesion from 51 abomasa were examined in histopathology. From them, three samples (5%) could not be clearly classified as a lesion or not. Of these three samples, one was macroscopically classified as an erosion and two as scars. Of the remaining 57 samples, 25, 2, and 29 were histologically identified as erosion, ulcer, and scar, respectively, and one lesion that was considered an erosion macroscopically was determined to be an erosion with a scar on histopathology. Examples of the histological presentation of an erosion, ulcer, and scar can be found in Figure 3. The comparison between the macroscopical and histological typing of abomasal lesions can be found in Table 4. Scars, using histopathology, were often considered to be ulcers macroscopically (11/28, 39%) and erosions, using histopathology, were sometimes seen as ulcers macroscopically (4/25, 16%).

## 4. Discussion

The prevalence of abomasal lesions in veal calves in Europe was reported as high, ranging from 73% to 100% [1,2]. Therefore, a high prevalence was expected in veal calves in Quebec as well. This study showed a prevalence of abomasal lesions of 100% in seven different groups of slaughtered calves, demonstrating the potentially high importance of this problem. The majority of the lesions were present in the pyloric area. This is in agreement with the previous literature [9,28,29], although lesions can also be found scattered throughout the abomasum [30] and in the fundic area [1,31]. Since there are only minor differences in milk-fed veal farm management between Europe and Canada, similarities regarding prevalence and lesion distribution was expected. Although the etiology of abomasal lesions is generally accepted as multifactorial [4], lesions in the fundic area are mostly attributed to stress, and lesions in the pyloric area to inadequate feeding [9,28,29,31]. Given the abundance of lesions in the pyloric area in this study, inadequate feeding should thus be suspected to play a major role, which is also acknowledged by the previous literature [4,32].

To our knowledge, the present study is the first to compare the inter-rater reliability of scoring systems for abomasal lesions in calves. In this study, a poor to very good agreement was seen for the pyloric area and torus pyloricus, although a higher agreement was observed when combining lesions in the pyloric area. For the fundic area, a poor to very good agreement was seen regarding the presence or absence of a lesion and a poor to moderate agreement regarding the number of lesions. This is in accordance with previous studies in horses, where reliable to moderate inter-rater agreement has been noted for gastric ulcer scoring systems [33,34,35]. Among these studies, one shows a good reliability of clinical scoring system for gastric ulcers in horses [35]. However, this system is ordinal, containing a score from 0 to 4. It is suggested that the more complex scoring systems are, the lower their reliability [35], although one study with a very simple scoring system (0–2) showed an unacceptable reliability agreement [34]. Due to this high variability in inter-rater agreement described in the literature [33,34,35] and shown in the present study, and in order to minimize misclassification, it is suggested that a detailed description of each score is provided in combination with sufficient photographic material [35,36].

When conducting a reliability study, a minimum of 30 samples and three raters is generally recommended [26], which was achieved in this study. Differentiation of the type of abomasal lesions was deemed difficult, since poor agreement was obtained at the location where most lesions occur, namely the pyloric area. When combining various types of lesions, agreement improves but remains insufficient. These results are supported by the histological examination performed in this study. Since the difference between erosion and an ulcer is defined by the perforation of the lamina muscularis mucosae [9,12,13], it seems plausible that errors are made macroscopically due to difficulties in determining if the lamina muscularis mucosae is perforated or not. Additionally, histologically confirmed scars can be as easily misclassified as acute ulcers macroscopically. When the typical star-shaped appearance is present, classification is easier. However, when only a small amount of fibrous tissue is present in the samples, this is histologically classified as a scar [8,12], but still appears to be an ulcer macroscopically. Additionally, a few macroscopically apparent lesions could not be defined as such by histology. Possible explanations for this are the biological variation in the color of the mucosae, given the impression of being a lesion-like erosion or ulcer, or the coincidental folding of the plica, giving the appearance of a scar. Regarding the fact that an erosion can develop into an ulcer, which can then become chronic and heal, thus evolving into a scar in time, one must question the added value of typing different kinds of lesions. Previous studies have suggested an increase in clinical impact depending on the severity of the lesion [30]. However, to our knowledge, this assumption has not been proven, except for perforated ulcers. Perhaps due to the difficulty of classifying lesions, a scoring system only accounting for the size and number of lesions in the pyloric region and torus pyloricus was developed [2] in accordance with the Welfare Quality Consortium protocol for veal calves in 2009. Regarding the absolute agreement between raters, a low ICC value was obtained, showing that the agreement between different raters was low for obtaining the same absolute number (i.e., the score given to an abomasum depends on the rater). Nevertheless, looking at the average agreement, an acceptable value was seen, showing that the severity ranking of the abomasum is comparable between raters. Thus, this scoring system can be used to determine the severity of lesions in general, allowing for the same overall ranking of low and high scores for abomasa for different raters.

The etiology of abomasal lesions in veal calves is considered to be complex and multifactorial. However, it is unknown if part of this apparent complexity is secondary to the absence of a gold standard method to assess abomasal lesions. This study shows that the macroscopical classification of abomasal lesions is challenging and may, per se, induce misclassification bias. This limitation may complexify studies that are attempting to identify risk factors associated with these lesions in the absence of a gold standard. This challenge is commonly observed in various medical fields and may interfere with study robustness [37].

A limitation of this study is that no intra-rater agreement, where the same rater scores the abomasa several times, was examined. However, since this study demonstrates that the score depends on the rater, new scoring systems and veterinary courses should be implemented regardless of the intra-rater agreement. We defined a priori different rater types that would potentially mimick the field application of a scoring system. In this study, two raters were senior researchers, whereas the two other raters were veterinary students (1st and 2nd year of veterinary cursus). The latter could be representative of future users of the score in slaughterhouse, as they are raters with a minimal scientific and anatomic background. Interestingly, a higher overall % of raw agreement could be seen between the pair of senior researchers (81.1%) compared to the pair of students (69.9%), indicating that experience can help improve the system, as previously indicated [35]. When a more reliable scoring system is developed, new studies could be implemented on a larger scale, making it possible to accurately determine possible risk factors of abomasal lesions in veal calves. A better understanding of this problem can hopefully help in developing new ante-mortem diagnostic techniques, which can support treatment and ultimately prevention.

## 5. Conclusions

When examining 76 abomasa retrieved from veal calves in the slaughterhouse in Quebec, at least one lesion was detected on all abomasa, demonstrating the high prevalence of this problem. A large variation in inter-rater agreement was demonstrated for current scoring systems of abomasal lesions, depending on the type of the lesion and on the location. Acceptable reliability was observed for the general ranking of the lesions across all abomasa which were scored. However, individual abomasum scoring of the type of lesion and localisation depends on the rater. Additionally, lesion type can be difficult to classify macroscopically compared to histological examination. These results suggest that the development and implementation of a new reliable scoring system can help to better understand this underestimated problem.

## Figures and Tables

**Table 1:** Prevalence (%) of lesions and distribution (median and range) of the number of lesions in affected abomasa, shown per location (fundic area, pyloric area, and torus pyloricus) and per type of lesion (erosion, ulcer, and scar). These data are obtained by macroscopical scoring of one observer, namely the veterinarian and PhD, of 76 abomasa of veal calves in Quebec.

Lesion	Localisation	No. (%) with ≥1 Lesion	Distribution of the Number of Lesions	
			Median (95% CI *)	Range
Erosion	Fundic area	13 (17.1%)	7 (2–17)	1–21
	Pyloric area	67 (88.2%)	7 (5–9)	1–60
	Torus pyloricus	25 (32.9%)	2 (1–3)	1–9
Ulcer	Fundic area	2 (2.6%)	1.5 (-)	1–2
	Pyloric area	26 (34.2%)	2 (1–3)	1–40
	Torus pyloricus	11 (14.5%)	1 (1–1)	1–4
Scar	Fundic area	1 (1.3%)	4 (-)	4
	Pyloric area	22 (28.9%)	1 (1–2)	1–4
	Torus pyloricus	4 (5.3%)	1 (-)	1

* Confidence interval; (-): No 95% confidence interval could be obtained.

**Table 2:** Heat-plot summarizing the Fleiss κ, Gwet’s agreement coefficient type 1 (AC1) and intra-class correlation coefficient (ICC) between four raters assessing abomasal lesions in veal calves at a representative slaughterhouse in Quebec, Canada. Dark green cells indicate excellent reliability, light green cells good reliability, yellow cells moderate reliability, light red cells slight reliability, and dark red cells poor reliability, according to previously reported guidelines [21,26]. ^a^ ICC-values were calculated for the number of lesions per type and location.

Grouping of Lesions	Lesion	Location	Fleiss Kappa	Gwet’s AC1	ICC ^a^
Single lesion	Erosion	Fundic area	0.70	0.90	0.52
		Pyloric area	0.03	0.46	0.23
		Torus pyloricus	0.33	0.39	0.39
	Ulcer	Fundic area	0.17	0.90	0.03
		Pyloric area	0.00	0.12	0.21
		Torus pyloricus	0.23	0.49	0.22
	Scar	Fundic area	0.50	0.97	0.73
		Pyloric area	0.34	0.48	0.55
		Torus pyloricus	0.12	0.83	0.11
Two lesions	Erosion and/or ulcer	Fundic area	0.76	0.89	0.66
		Pyloric area	0.09	0.93	0.46
		Torus pyloricus	0.39	0.41	0.42
	Ulcer and/or scar	Fundic area	0.32	0.87	0.11
		Pyloric area	0.09	0.43	0.23
		Torus pyloricus	0.32	0.46	0.22
	Erosion and/or scar	Fundic area	0.69	0.87	0.52
		Pyloric area	0.12	0.68	0.23
		Torus pyloricus	0.37	0.38	0.39
Any lesions	Erosion and/or ulcer and/or scar	Fundic area	0.76	0.88	0.66
		Pyloric area	0.14	0.95	0.45
		Torus pyloricus	0.48	0.52	0.42

**Table 3:** Raw percentage of agreement (Pa), Cohen’s *κ* and Gwet’s agreement coefficient type 1 (AC1) to compare different pairs of raters, shown per different type of lesion and per localisation. Raters 1 and 3 are unexperienced veterinary students, whereas raters 2 and 4 are considered senior and experienced.

	Lesion	Erosion			Ulcer			Scar			
	Localisation	Fundic Area	Pyloric Area	Torus Pyloricus	Fundic Area	Pyloric Area	Torus Pyloricus	Fundic Area	Pyloric Area	Torus Pyloricus	Average
Rater 1_2	Pa	94.7	65.8	64.5	92.1	43.4	73.7	96.1	63.2	82.9	75.4
	Cohen’s kappa	0.81	0.16	0.28	0.37	0.076	0.27	0.39	0.19	0.061	0.29
	Gwet’s AC1	0.93	0.45	0.32	0.91	−0.09	0.60	0.96	0.33	0.79	0.58
Rater 1_3	Pa	94.7	59.2	68.4	85.5	78.9	75	96.1	71.1	78.9	69.9
	Cohen’s kappa	0.81	0.11	0.36	0.079	0.35	0.43	0.55	0.40	0.15	0.36
	Gwet’s AC1	0.93	0.25	0.38	0.83	0.69	0.56	0.96	0.44	0.72	0.64
Rater 1_4	Pa	88.2	57.9	69.7	88.2	53.9	71.1	96.1	63.2	84.2	74.7
	Cohen’s kappa	0.51	−0.02	0.39	0.13	−0.037	0.31	0.39	0.16	−0.025	0.20
	Gwet’s AC1	0.85	0.33	0.41	0.86	0.23	0.50	0.96	0.36	0.82	0.59
Rater 2_3	Pa	97.4	67.1	59.2	93.4	46.1	72.4	97.4	71.1	90.8	77.2
	Cohen’s kappa	0.91	0.08	0.14	0.26	0.068	0.29	0.49	0.38	0.49	0.35
	Gwet’s AC1	0.96	0.50	0.23	0.93	−0.07	0.56	0.97	0.47	0.89	0.60
Rater 2_4	Pa	90.8	81.6	68.4	93.4	47.4	68.4	100	86.8	93.4	81.1
	Cohen’s kappa	0.62	0.12	0.30	−0.033	−0.0026	0.12	1	0.65	−0.022	0.31
	Gwet’s AC1	0.88	0.77	0.43	0.93	−0.05	0.52	1	0.79	0.93	0.69
Rater 3_4	Pa	88.2	61.8	77.6	92.1	48.7	56.6	97.4	71.1	84.2	75.3
	Cohen’s kappa	0.51	−0.06	0.53	0.21	−0.11	0.0079	0.49	0.36	−0.025	0.21
	Gwet’s AC1	0.85	0.42	0.57	0.91	0.07	0.23	0.97	0.49	0.82	0.59

**Table 4:** Comparison between macroscopical and histological typing of lesions of 60 tissue samples retrieved from abomasa of veal calves in the slaughterhouse.

		Macroscopical Typing			
		Erosion	Ulcer	Scar	Total
Histological typing	Erosion	19	4	2	25
	Ulcer	1	1	/	2
	Scar	1	11	17	29
	Erosion + Scar	1	/	/	1
	Unknown	1	/	2	3
	Total	23	16	21	60

## Data Availability

Publicly available datasets were analyzed in this study. This data can be found in Supplementary Materials.

## Supplementary Materials

The following supporting information can be downloaded at: https://www.mdpi.com/article/10.3390/ani13101664/s1, Supplementary Data.
