# Supplementary material for: Inter-Rater Reliability of Scoring Systems for Abomasal Lesions in Quebec Veal Calves
Source: Animals (Basel). 2023 May 17;13(10):1664. doi: 10.3390/ani13101664 (PMC10215123; doi:10.3390/ani13101664)
Supplement: Supplementary file 1 [file animals-13-01664-s001.zip › animals-2354923-supplementary.docx]

Supplementary data

title: "Abomasal ulcers"

author: "Laura Van Driessche"

date: "2022-09-08"

output:

word_document: default

html_document: default

pdf_document: default

---

## Dataset with only abomasa with a torus pylorus: removed ID 13,20,23,26,52

##library

```{r setup, include=FALSE}

library(tidyverse)

library(readxl)

library(dplyr)

library(gapminder)

library(haven)

library(readr)

library(janitor)

library(socviz)

library(here)

library(stringr)

library(purrr)

library(srvyr)

library(visdat)

library(broom)

library(printy)

library(usethis)

library(cowplot)

library(gghighlight)

library(colorspace)

library(palmerpenguins)

library(naniar)

library(FSA)

library(FactoMineR)

library(factoextra)

library(irr)

library(irrCAC)

library(psych)

# 1. Agreement and ICC per lesion per location

```{r}

df <- data_herwerkt_torus %>% select(ID, "portion glandulaire fundique", "portion pylorique",

"torus pylorique", "érosion", "ulcère", "cicatrice",

"nombre de lésion", "observateur" ) %>%

rename( "Fund"="portion glandulaire fundique", "Pyl"= "portion pylorique", "Tor"="torus pylorique",

"Eros"="érosion", "Ulc"= "ulcère", "Cic"="cicatrice", "N_les"="nombre de lésion", "Obs"= "observateur")

df$ID=as.numeric(df$ID)

#dput(colnames(df))

str(df)

df <- df %>% mutate_at(c('N_les', 'Eros', 'Ulc', 'Cic'), as.numeric)

df <- df %>% mutate(Obs=as.factor(Obs))

levels(df$Obs)

df <- df %>% pivot_longer(cols=c("Fund", "Pyl", "Tor"), names_to = "Site")

df <-df%>% arrange(ID, Obs)

head(df, n=25)

df <- df%>%

filter(value=="1")

df_pyl_eros <- df %>%

filter(Site=="Tor") %>% filter(Cic=="1") %>%

aggregate(N_les ~ ID+Site+Obs+Cic, ., sum)%>%

arrange(ID, Obs)

df_pyl_eros$ID=as.numeric(df_pyl_eros$ID)

df_pyl_eros <- df_pyl_eros %>% complete(., ID,Site, Obs) %>%

filter(!Obs%in%c("Op5", "Op6"))%>%

replace(., is.na(.), 0)

dfpyl <- df_pyl_eros

attach(dfpyl)

base <- Basic_format

attach(base)

total <- left_join(base, dfpyl)%>%

relocate(ID:N_les,.after = ID)

total <- total%>%

complete(., ID, Obs)%>%

filter(!Obs%in%c("Op5", "Op6"))

total <- total%>%

filter(Obs%in%c("Op1", "Op2", "Op3", "Op4"))%>%

select(ID, Obs, Cic, N_les)%>%

replace(., is.na(.), 0)

total <- total%>%

filter(!ID%in%c(13,20,23,26,52))

Kappa_pyl_Eros <- total %>%

select(ID, Obs, Cic) %>%

pivot_wider(names_from = Obs, values_from = Cic, values_fn = max) %>%

select(-ID)

ICC_pyl_Nles <- total %>%

select(ID, Obs, N_les) %>%

pivot_wider(names_from = Obs, values_from = N_les, values_fn = max) %>%

select(-ID)

agree(Kappa_pyl_Eros)

kappam.fleiss(Kappa_pyl_Eros)

ICC(ICC_pyl_Nles)

gwet.ac1.raw(Kappa_pyl_Eros)

# 2. Agreement and ICC per location with a combination of lesions

df_pyl_erulc <- df %>% select(ID, Ulc, Cic, N_les, Obs, Site) %>%

filter(Site=="Tor") %>% filter(Cic|Ulc=="1") %>%

aggregate(N_les ~ID+Site+Obs, ., sum)%>%

arrange(ID)

df_pyl_erulc$Les = les

df_pyl_erulc$ID=as.numeric(df_pyl_erulc$ID)

df_pyl_erulc <- df_pyl_erulc %>% complete(., ID,Site, Obs) %>%

filter(!Obs%in%c("Op5", "Op6"))%>%

replace(., is.na(.), 0)

dfpylerulc <- df_pyl_erulc

attach(dfpylerulc)

base <- Basic_format

attach(base)

total <- left_join(base, dfpylerulc)%>%

relocate(ID:N_les,.after = ID)

total <- total%>%

complete(., ID, Obs)%>%

filter(!Obs%in%c("Op5", "Op6"))

total <- total%>%

filter(Obs%in%c("Op1", "Op2", "Op3", "Op4"))%>%

select(ID, Obs, Les, N_les)%>%

replace(., is.na(.), 0)

total <- total%>%

filter(!ID%in%c(13,20,23,26,52))

Kappa_pyl_Erulc <- total %>%

select(ID, Obs, Les) %>%

pivot_wider(names_from = Obs, values_from = Les, values_fn = max) %>%

select(-ID)

ICC_pyl_Nles <- total %>%

select(ID, Obs, N_les) %>%

pivot_wider(names_from = Obs, values_from = N_les, values_fn = max) %>%

select(-ID)

agree(Kappa_pyl_Erulc)

kappam.fleiss(Kappa_pyl_Erulc)

ICC(ICC_pyl_Nles)

gwet.ac1.raw(Kappa_pyl_Erulc)
